# Shooting Scheme for Perturbations in Optimised Solution of the Orbital Boundary Value Problem

**DOI:** 10.1007/s42496-024-00246-0

**Published:** 2025-01-07

**Authors:** Alessandro Vananti, Harleen Kaur Mann, Thomas Schildknecht

**Affiliations:** Astronomical Institute, University of Bern, Sidlerstrasse 5, 3012 Bern, Switzerland

**Keywords:** Space debris, Orbit determination, Shooting method, Tracklet correlation

## Abstract

Monitoring of the near-Earth space environment has become more and more important in recent times. The constantly increasing amount of space debris is a major threat for space activities and an exhaustive knowledge about the space objects population is of primary importance for civil and military applications. The *surveillance* of the space surrounding the Earth is achieved by means of sensors based on different technologies. Passive optical observations using ground telescopes are usually employed to track space objects in high altitude orbits. The obtained measurements are sparse, and their arcs are very short. The computation of the orbit based on these observations is challenging and several methods have been proposed. One of the known approaches relies on an optimization scheme of the classical boundary value problem. However, in the latter, the object motion is described by a Keplerian orbit without considering additional perturbations. In this novel approach, orbital perturbations are introduced in the computation procedure using a shooting model. The improved algorithm is applied to simulated observations of objects in the geostationary region under the influence of solar radiation pressure. The measured arcs are separated by intervals of one or more orbit revolutions. The performance of the proposed method is evaluated in terms of accuracy considering different levels of perturbation.

## Introduction

The better understanding of the near-Earth space environment and a better control of possible threats are essential for the future of space activities. The increasing population of space debris poses a serious threat to spacecraft in orbit. The concept of surveillance applied to the space environment is therefore a necessary measure to avoid the worsening of the current situation. Space surveillance addresses different aspects related to space operations like mission support, collision avoidance, contingencies, fragmentation detections, and space traffic intelligence. These functions as well as the validation and improvement of space environment models require regular monitoring of the space objects population. To accomplish this, observations provided by networks of sensors are processed and used to build-up and maintain catalogues of orbital elements and additional characteristics. Today, statistically sufficient data are acquired by conducting optical surveys and by radar beam-park experiments, whilst novel observation techniques, such as laser ranging, are under development. The characterisation of the detected objects provides further input to both, the cataloguing and the modelling task. Such information may comprise the estimation of physical properties, such as, e.g., area-to-mass ratio, colour, tumbling state, and attitude change rates. For monitoring purposes, observations of the objects covering longer time intervals, in the order of multiple orbital revolutions, must be guaranteed to provide reliable information. The build-up of a catalogue and its maintenance depends on the capacity to determine the orbit of the observed objects from few measurements. In fact, only a limited number of observations are available per night per object, each over observation arcs that can be as short as a few seconds. Therefore, a single track, regardless of measurement type, often does not contain sufficient information to reliably estimate the observed object’s state or conduct follow-up observations. The sparse and short sequences of observations, called *tracklets*, need to be correlated or linked with each other in the so-called *tracklet correlation* [1]. To date, various methods have been developed to approach the tracklet association problem. The general strategy implies the computation of a hypothetical orbit in common for the two tracklets to be correlated. If an orbit exists that matches all observations well, it means that the tracklets belong to the same object and the correlation is successful. The methods differ in the way the problem is represented, i.e., using the initial value or the boundary value formulation, and on the type of observations available, i.e., angles-only or range measurements. In general, the solution space of the problem can be restricted to a so-called *admissible region*, originally formulated for semimajor axis of the orbit [2] and eccentricity [3]. A common approach is also to reduce the observations in a compressed representation, so-called *attributable*, introduced first in [4].

In the case of radar observations, where ranges and angles are available, initial orbits can be directly derived from the tracklets to be correlated using known orbit determination methods, e.g., Gooding’s solution of the Lambert problem [5], or the Goddard Trajectory Determination System (GTDS) range and angles method [6]. The orbits are then propagated to a common epoch and the matching is evaluated [7, 8]. Alternatively, a boundary value approach can be used as in [9] and [10], where the tracklets are represented by attributables. Related to radar but with other observables, in [11], a correlation method using angular and range rate measurements provided by Doppler-only radar is proposed.

With angles-only measurements using passive optical sensors, several approaches scan the admissible region for hypotheses in the attributable space. In the initial value formulation, the hypotheses in range and range rate at the initial epoch are sampled and propagated to the epoch of the attributable to be matched [12] or the intersection at a common epoch of two hyperplanes related to the attributables is determined [13]. Other methods are based on a boundary value scheme where range hypotheses at two tracklet epochs are used to compute an orbit common to the two attributables and its matching is considered to confirm the correlation. In this case, the range hypotheses combined with the angular measurements build a Lambert’s problem that can be solved with known algorithms [5, 14]. The solution in the range space can be found with a grid search [15, 16], with an optimised searching scheme [17, 18], or as in [19] using an analytical formulation based on the Keplerian integrals.

The tracklet association problem can be extended to three or more tracklets, to a so-called Multiple Target Tracking (MTT) problem. A thorough discussion of the different approaches to solve this problem and the difficulties encountered can be found in [20]. To optimally solve the MTT problem, all the possible combinations of observations have to be evaluated. When the dimension is larger than two (with two tracklets), the number of possible combinations rapidly increases and the problem is said to become Non-deterministic Polynomial-time (NP) hard. A popular and well-known algorithm is the Multiple Hypothesis Tracking (MHT) algorithm [21]. Other approaches are based on probabilistic models [22–24], or Markov Chain Monte Carlo (MCMC) computations [25]. NP-hard problems are sometimes solved adopting evolutionary algorithms. In [26], a procedure using a genetic algorithm is employed for multiple tracklets association.

The initial orbit obtained from the tracklet correlation algorithm is usually refined with a batch least-squares or a sequential estimator to use all the available observations, to include orbit perturbations, and to reduce the number of false alarms in the correlation problem [7, 24]. In the framework of differential algebra, the pruning of wrong correlations with a least-squares procedure is analysed in [27], whilst, in [28], the performance of a new correlation algorithm is presented. In [29], an algorithm is proposed that combines both initial orbit determination and orbit refinement with an arbitrary number of observations and that can be successfully applied for tracklet association.

In the present paper, we focus on the method for passive optical observations presented in [17], which follows a boundary value problem formulation. This approach is sometimes referred as Optimised Boundary Value Initial Orbit Determination (OBVIOD) method [26]. The algorithm determines the ranges at initial and final epoch with an optimization procedure. In the iteration scheme, the orbit is computed with a Lambert solver using the ranges and angular values at the boundary. However, the Lambert solution usually considers only pure Keplerian orbits without the effect of perturbations. In this work, we propose a novel algorithm based on a *shooting* approach that includes perturbations in the OBVIOD method. Simulated passive optical measurements in a typical survey scenario for geostationary regime are generated to test the new algorithm. Results with and without the perturbation effect of the solar radiation pressure are compared. The accuracy of the computed orbits is assessed with different values in: observation noise, interval between tracklets, eccentricity of the object orbit, and magnitude of the perturbing force.

## Description of Boundary Value Method

Passive optical observations assumed in the context of the correlation problem are in form of *tracklets*, short-arc series of topocentric measurements in right ascension $\alpha$ and declination $\delta$, typically several seconds to few minutes long. Observations within a single tracklet provide in addition to the explicit angles also the angular rate that can be derived from the time series. The *attributable* is the compressed form of a tracklet at the epoch *t*

1 $$A_{t} = \left( {\alpha ,\dot{\alpha },\delta ,\dot{\delta }} \right)_{t} .$$

We assume here that the angular rates are computed through a linear regression, although other fitting procedures have also been proposed e.g., in [9]. The measurement noise ${\upsigma }_{obs}$ results in the following approximate variance for attributable angles $\theta$ and angle rates $\dot{\theta }$, given $\Delta t$ as the interval between $N$ observations [12]:

$${\sigma }_{\theta }^{2}=\frac{1}{N}{\sigma }_{\text{obs}}^{2}$$

2 $${\sigma }_{\dot{\theta }}^{2}=\frac{12}{{\Delta t}^{2}N}{\sigma }_{\text{obs}}^{2}.$$

Following [17], the classical boundary value problem is formulated with the angular positions ${\alpha }_{\text{1,2}},{\delta }_{\text{1,2}}$ at the boundary epochs ${t}_{1}, {t}_{2}$. The orbit is uniquely defined with hypothetical topocentric ranges ${\rho }_{1}, {\rho }_{2}$. These angles and ranges characterise a typical Lambert problem, where in general two position vectors and the time between them are given. From the Lambert solution with the hypothetical ranges, the angular rates $\dot{\alpha }_{1,2}^{\prime } ,\dot{\delta }_{1,2}^{\prime }$ are computed. The matching quality between the computed $\dot{\alpha }_{1,2}^{\prime } ,\dot{\delta }_{1,2}^{\prime }$ and the observed ${\dot{\alpha }}_{\text{1,2}},{\dot{\delta }}_{\text{1,2}}$ is quantified as Mahalanobis distance

3 $${d}_{\text{M}}=\sqrt{{\left(\dot{{\varvec{z}}}-\dot{\varvec{z}}^{\prime}\right)}^{\text{T}}{{\varvec{C}}}^{-1}\left(\dot{{\varvec{z}}}-\dot{\varvec{z}}^{\prime}\right)},$$

where $\dot{{\varvec{z}}}=\left({\dot{\alpha }}_{1},{\dot{\delta }}_{1},{\dot{\alpha }}_{2},{\dot{\delta }}_{2}\right)$, $\dot{\varvec{z}}^{\prime}=\left(\dot{\alpha}_{1}^{\prime},\dot{\delta}_{1}^{\prime},\dot{\alpha}_{2}^{\prime},\dot{\delta}_{2}^{\prime}\right)$, and ${\varvec{C}}={\varvec{C}}_{\dot{\varvec{z}}}+{\varvec{C}}_{\dot{\varvec{z}}^{\prime}}$ is the sum of the covariance matrices related to $\dot{{\varvec{z}}}$ and $\dot{\varvec{z}}^{\prime}$.

The orbital solution of the boundary value problem is searched as the minimum of the distance function ${d}_{\text{M}}$ in the space of hypothetical ranges ${\rho }_{1}, {\rho }_{2}$. The search can be performed with an optimization algorithm, e.g., following a Broyden–Fletcher–Goldfarb–Shanno (BFGS) procedure. If the minimal distance is below a certain threshold, it means that the angular rates of both tracklets are compatible with the found solution. This is also a confirmation of the tracklet correlation, namely that the two tracklets belong to the same object. If we consider normal distributed angular rates, the statistical distribution of ${d}_{\text{M}}^{2}$ follows a chi-squared distribution ${\chi }_{k}^{2}$ with $k=2$, corresponding to two degrees of freedom [26]. This allows the definition of a threshold value for the required confidence level by evaluating its quantile function. In general, the computed values of the latter with different degrees of freedom can be found in reference tables. Specifically in this case with $k=2$, the ${\chi }_{2}^{2}$ distribution reduces to an exponential function and can be evaluated in closed form. As in other methods, also here the search space can be reduced to an *admissible region*. We define the position of the observed object in a geocentric frame as ${\varvec{r}}=\boldsymbol{ }{{\varvec{r}}}_{s}+{\varvec{\rho}}$, where ${{\varvec{r}}}_{s}$ is the position of the observing sensor and ${\varvec{\rho}}$ is the line of sight vector. The admissible region can be confined between the topocentric ranges ${\rho }_{min}$ and ${\rho }_{max}$ [17], which are computed using

4 $${\rho }_{\text{min}/\text{max}}=-c+\sqrt{{c}^{2}+{r}_{\text{min}/\text{max}}^{2}-{r}_{s}^{2}},$$

where $c={\varvec{r}}_{s}\cdot{\varvec{\rho}}$. We compute ${r}_{\text{min}}={a}_{\text{min}}(1-{e}_{\text{max}})$ and ${r}_{\text{max}}={a}_{\text{max}}(1+{e}_{\text{max}})$ using minimal and maximal values for semimajor axis ${a}_{\text{min}/\text{max}}$ and eccentricity ${e}_{\text{max}}$ that can be selected depending on the considered orbital region [17].

## Lambert Problem

One important step in the above iteration procedure is the solution of the Lambert problem. In the following, we refer to the work of Izzo [14], which is based on [5] and [30]. We consider two position vectors ${{\varvec{r}}}_{1}$ and ${{\varvec{r}}}_{2}$ and the time of flight $t$. The transfer angle from ${{\varvec{r}}}_{1}$ to ${{\varvec{r}}}_{2}$ is defined as $\theta \in \left[0, 2\pi \right]$. Depending on whether $\theta \in \left[0, \pi \right]$ or $\theta \in \left[\pi , 2\pi \right]$ a different path between the two positions is described corresponding to either a prograde or a retrograde orbit. In general, the information about the sense of rotation is available according to the observed class of objects and the specific orbital region. To compute the orbit, it is useful to introduce the parameter

5 $$\lambda =\frac{\sqrt{{r}_{1}{r}_{2}}}{s}\text{cos}\frac{\theta }{2}=\pm \sqrt{\frac{s-c}{s}},$$

where $s=\frac{1}{2}\left({r}_{1}+{r}_{2}+c\right)$ is the semiperimeter of the triangle with sides ${r}_{1}$, ${r}_{2}$, and $c=\left|{{\varvec{r}}}_{1}-{{\varvec{r}}}_{2}\right|$. The parameter $\lambda \in \left[-1, 1\right]$ is positive when $\theta \in \left[0, \pi \right]$ and negative when $\theta \in \left[\pi , 2\pi \right]$. Note that $\lambda$ is an invariant of the Lambert problem and all the triangles with equal $\lambda$ belong to the same class of equivalence. This allows us to solve the problem in a reduced parameter space. The dynamics of the problem is implicit in the time of flight $t$ and in the energy of the orbit, related to the semimajor axis $a$. It is beneficial to define the non-dimensional time of flight

6 $$T=\frac{1}{2}\sqrt{\frac{\mu }{{a}_{m}^{3}}}t,$$

where ${a}_{m}=s/2$ is the semimajor axis of the minimum energy orbit. We also define the quantity $x$ satisfying

7 $${x}^{2}=1-\frac{s}{2a}.$$

For elliptic orbits $\left|x\right|<1$, whilst for the parabolic and hyperbolic case, $x=1$ and $x>1$, respectively. The situation with $x=$ 0 corresponds to the minimum energy orbit. In the following we consider only elliptic orbits. We see that for given $s$ and $a$, there are two possible values of $x$. These are referred as high path, with $-1<x<0$, and low path, with $0<x<1$. It can be shown that

8 $$T=\frac{1}{2}\sqrt{\frac{{a}^{3}}{{a}_{m}^{3}}}\left(\left(\alpha -\text{sin}\alpha \right)-\left(\beta -\text{sin}\beta \right)+2\pi N\right),$$

where $N$ is the number of orbital revolutions in the transfer and the auxiliary angles $\alpha$ and $\beta$ are defined through

9 $$\frac{s}{2a}={\text{sin}}^{2}\frac{\alpha }{2}$$

10 $$\frac{s-c}{2a}={\text{sin}}^{2}\frac{\beta }{2}.$$

**Fig. 1:**
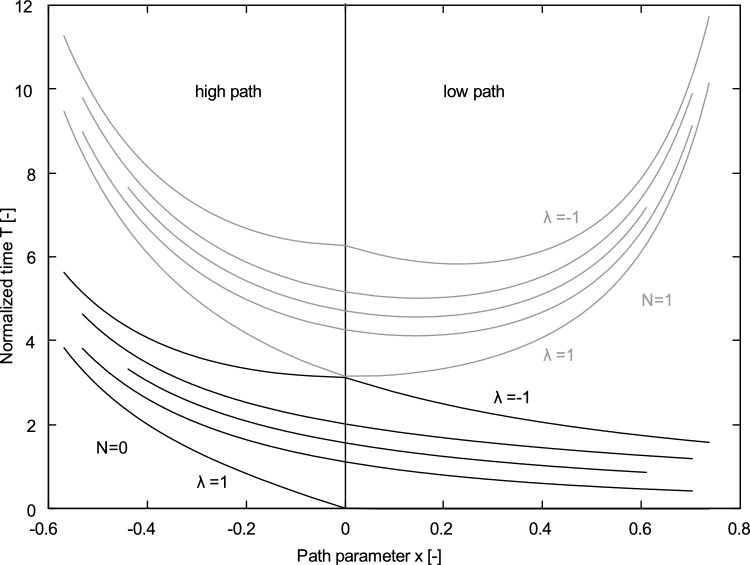
Normalised time $T$ as a function of $x$ for different $\lambda$ values ($\lambda =$ 1, 0.8, 0, -0.8, -1)

It is possible to plot $T$ as a function of $x$ for a given $\lambda$. Figure 1 shows examples of the $T$ function with different values of $\lambda$, for the case below one orbital revolution ($N$ = 0) and between one and two revolutions ($N$ = 1). The diagram is divided into two parts (high and low path) by the minimum energy orbit line. It covers only values of $x$ within the interval for elliptic orbits ($\left|x\right|<1$). In general, for $N$ = 0, the function also describes hyperbolic orbits and continues in the range $x>1$. For an exhaustive illustration of the different regimes, see e.g., [14]. In the case $N$ = 1 for a given time of flight, respectively $T$, and $\lambda$, we see from the diagram that there are two possible intersections, or solutions for $x$. Note that there is not always one high and one low path solution, since the minimum of the function does not coincide with the minimum energy line. This is also the case for higher numbers of revolutions $N$ > 0. Thus, in general for the multirevolution Lambert’s problem, even if the sense of the orbital motion (prograde/retrograde) and the number of revolutions is known, the solution is not unique. One of the two existing solutions needs to be discarded according to additional information about the orbit, e.g., a component of the velocity at the boundary epoch.

## Shooting Method

A general approach to take into account perturbations in orbital problems is to include the additional accelerations in the numerical integration procedure used to solve the equations of motion. In the case of a two point boundary value problem, two ways can be considered: shooting methods and relaxation methods [31]. In the relaxation method, the differential equations are replaced by finite-difference equations on a mesh of points. The selection of the points and their distances is specific to the problem and has to be defined according to the characteristic of the differential equations. The solution is found adjusting the values on the mesh to satisfy the differential equations and the boundary conditions. If number and position of the required points are not known a priori, shooting methods are usually preferred. In the latter, hypothetical initial values are chosen as free parameters at one boundary and, after integration up to the second boundary, the end values are compared with the boundary conditions. The solution is found minimising the discrepancy at the second boundary.

The minimization in the shooting method can be solved with a classical Newton–Raphson algorithm or other existing optimization scheme. In the context of orbital problems, for example, also Gauss–Newton [32] and Bisection method [33] were adopted. Several variations of the basic shooting method were proposed in the literature. If the function has a problematic convergence and it is difficult to find an appropriate starting point, homotopy continuation methods can be applied [34]. In [35], the *method of particular solutions* is studied to improve the speed of the iterative search of the solution. Recently, algorithms based on differential algebra [36] and on the theory of functional connections [37] were proposed.

The basic application of the shooting approach is referred as *single shooting*. The equations of motion of the orbital problem can be written as first-order differential equations

11 $$\dot{{\varvec{x}}}\left(t\right)={\varvec{u}}\left(t, {\varvec{x}}\left(t\right),{\varvec{\lambda}}\right),$$

where ${\varvec{x}}\left(t\right)$ is the state vector with positions and velocities, and ${\varvec{\lambda}}$ represents possible additional parameters. Given an initial value ${\varvec{x}}\left({t}_{0}\right)={{\varvec{x}}}_{0}$ we can integrate Eq. (11) and compute ${\varvec{x}}\left({t}_{1}\right)={{\varvec{x}}}_{1}$ at a later time ${t}_{1}$. In the shooting procedure, the initial value ${{\varvec{x}}}_{0}$ is considered as optimization variable to satisfy the condition at ${t}_{1}$. Specifically, if e.g., position vectors ${{\varvec{r}}}_{0}({t}_{0})$ and ${{\varvec{r}}}_{1}({t}_{1})$ define the boundary conditions, as in the Lambert problem, the shooting iteration searches for the appropriate initial velocity vector ${{\varvec{v}}}_{0}({t}_{0})$ that satisfies the condition at ${t}_{1}$. At every iteration, a state vector with given position ${{\varvec{r}}}_{0}$ and hypothetical velocity ${{\varvec{v}}}_{0}$ is propagated to the time ${t}_{1}$ and compared with the expected boundary condition ${{\varvec{r}}}_{1}$.

A possible extension of the shooting procedure is *multiple shooting*. Here, the path between two boundaries at epochs ${\tau }_{0}$ and ${\tau }_{n}$ is divided into small arcs ${{\varvec{x}}}_{j}(t)$ that connect multiple nodes ${{\varvec{x}}}_{j}\left({\tau }_{j}\right)$ at epochs ${\tau }_{j}$ where ${\tau }_{0}<{\tau }_{1}<\dots {<\tau }_{n}$. Additional conditions are necessary to ensure the continuity of the overall function ${\varvec{x}}\left(t\right)$ at the nodes: ${{\varvec{x}}}_{j}\left({\tau }_{j+1}\right)={{\varvec{x}}}_{j+1}\left({\tau }_{j+1}\right)$. Having multiple nodes, the boundary conditions do not need necessarily to be at ${\tau }_{0}$ and ${\tau }_{n}$. Note that, in general, also the times ${\tau }_{j}$ can be considered as free parameters, but for our problem we will only focus on the case with conditions at given start and final epoch. Multiple shooting has the advantage of a more reliable convergence in those cases where the initial guess for the trajectory is not so good. Moreover, a priori information from e.g., additional measurements can be included at multiple nodes in the initial guess.

We follow the implementation in [38] and define ${\varvec{X}}={\left({{\varvec{x}}}_{0},\boldsymbol{ }\dots {,{\varvec{x}}}_{\text{n}}\right)}^{\text{T}}$ and the constraint vector

12 $${\varvec{F}}\left({\varvec{X}}\right)=\left[\begin{array}{c}{{\varvec{x}}}_{0}\left({\tau }_{1}\right)-{{\varvec{x}}}_{1}\left({\tau }_{1}\right)\\ \vdots \\ {{\varvec{x}}}_{n-1}\left({\tau }_{n}\right)-{{\varvec{x}}}_{n}\left({\tau }_{n}\right)\\ {\varvec{f}}({{\varvec{x}}}_{0}\left({\tau }_{0}\right),{{\varvec{x}}}_{n}\left({\tau }_{n}\right))\end{array}\right]=0.$$

The conditions ${\varvec{f}}\left({{\varvec{x}}}_{0},{{\varvec{x}}}_{n}\right)=0$ are the original conditions at the boundary ${{\varvec{x}}}_{0,r}\left({\tau }_{0}\right)={{\varvec{r}}}_{0}$ and ${{\varvec{x}}}_{n,r}\left({\tau }_{n}\right)={{\varvec{r}}}_{n}$, where the suffix $r$ specifies that only the position components of ${{\varvec{x}}}_{i}$ are considered. For the multi-variable Newton method, we calculate the Jacobian matrix

13 $$\left(\frac{\partial {\varvec{F}}\left({\varvec{X}}\right)}{\partial {\varvec{X}}}\right)=\left[\begin{array}{ccccc}\frac{\partial {{\varvec{x}}}_{0}\left({\tau }_{1}\right)}{\partial {{\varvec{x}}}_{0}\left({\tau }_{0}\right)}& \frac{\partial {{\varvec{x}}}_{1}\left({\tau }_{1}\right)}{\partial {{\varvec{x}}}_{1}\left({\tau }_{1}\right)}& 0& 0& 0\\ 0& \frac{\partial {{\varvec{x}}}_{1}\left({\tau }_{2}\right)}{\partial {{\varvec{x}}}_{1}\left({\tau }_{1}\right)}& -\frac{\partial {{\varvec{x}}}_{2}\left({\tau }_{2}\right)}{\partial {{\varvec{x}}}_{2}\left({\tau }_{2}\right)}& 0& 0\\ 0& 0& \ddots & \ddots & \boldsymbol{ }\\ 0& 0& 0& \frac{\partial {{\varvec{x}}}_{n-1}\left({\tau }_{n}\right)}{\partial {{\varvec{x}}}_{n-1}\left({\tau }_{n-1}\right)}& -\frac{\partial {{\varvec{x}}}_{n}\left({\tau }_{n}\right)}{\partial {{\varvec{x}}}_{n}\left({\tau }_{n}\right)}\\ \frac{\partial {\varvec{f}}\left({{\varvec{x}}}_{0},{{\varvec{x}}}_{n}\right)}{\partial {{\varvec{x}}}_{0}\left({\tau }_{0}\right)}& 0& 0& 0& \frac{\partial {\varvec{f}}\left({{\varvec{x}}}_{0},{{\varvec{x}}}_{n}\right)}{\partial {{\varvec{x}}}_{n}\left({\tau }_{n}\right)}\end{array}\right],$$

which can be written as

14 $$\left(\frac{\partial {\varvec{F}}\left({\varvec{X}}\right)}{\partial {\varvec{X}}}\right)=\left[\begin{array}{cccc}{\boldsymbol{\Phi }}_{1}& -{{\varvec{I}}}_{6\text{x}6}& & \\ & \ddots & \ddots & \boldsymbol{ }\\ \boldsymbol{ }& \boldsymbol{ }& {\boldsymbol{\Phi }}_{n-1}& -{{\varvec{I}}}_{6\text{x}6}\\ {{\varvec{F}}}_{1}& 0& 0& {{\varvec{F}}}_{n}\end{array}\right],$$

where ${\boldsymbol{\Phi }}_{j}={\boldsymbol{\Phi }}_{j}({\tau }_{j},{\tau }_{j+1})$ is the state transition matrix for the ${{\varvec{x}}}_{j}(t)$ arc, ${\varvec{I}}$ is the unit matrix, whilst

15 $${{\varvec{F}}}_{0}=\left[\begin{array}{cc}{{\varvec{I}}}_{3\text{x}3}& 0\\ 0& 0\end{array}\right]\text{and }{{\varvec{F}}}_{n}=\left[\begin{array}{cc}0& 0\\ {{\varvec{I}}}_{3\text{x}3}& 0\end{array}\right].$$

The Newton iteration scheme follows the first-order update equation:

16 $${{\varvec{X}}}^{j+1}={{\varvec{X}}}^{j}-{\left(\frac{\partial {\varvec{F}}\left({{\varvec{X}}}^{j}\right)}{\partial {{\varvec{X}}}^{j}}\right)}^{-1}{\varvec{F}}\left({{\varvec{X}}}^{j}\right),$$

where ${{\varvec{X}}}^{j}$ and ${{\varvec{X}}}^{j+1}$ are current and next iteration in the scheme. The solution is reached when the ${L}^{2}$ norm of the constraint vector $\left|{\varvec{F}}\left({\varvec{X}}\right)\right|$ is below a specified convergence tolerance. In the present work a tolerance of 1.0 was set. The above algorithm is implemented in the Orbit Extrapolation Kit (Orekit) library [39].

## Algorithm for Boundary Value Problem

Based on the shooting method and the considerations on the Lambert problem, we want to solve the boundary value problem described in Sect.  2 in the presence of perturbations. The idea is to use the same underlying principle with the optimised search in the $({\rho }_{1}, {\rho }_{2})$ space and to replace the Lambert solver with a shooting iteration. The main reason is that the topography of the loss function to be minimised in the $({\rho }_{1}, {\rho }_{2})$ space is smooth and allows a searching algorithm to converge to a unique minimum. This is an advantage compared to a search, e.g., in the $({\rho }_{1}, {\dot{\rho }}_{1})$ space, where the topography presents several local minima [17]. According to [18], caution is advised when the time interval between the tracklets is a multiple of the orbital period. Furthermore, convergence to a unique point is only guaranteed with a proper separation of the multiple solutions occurring in the multi-revolutions Lambert problem.

**Fig. 2:**
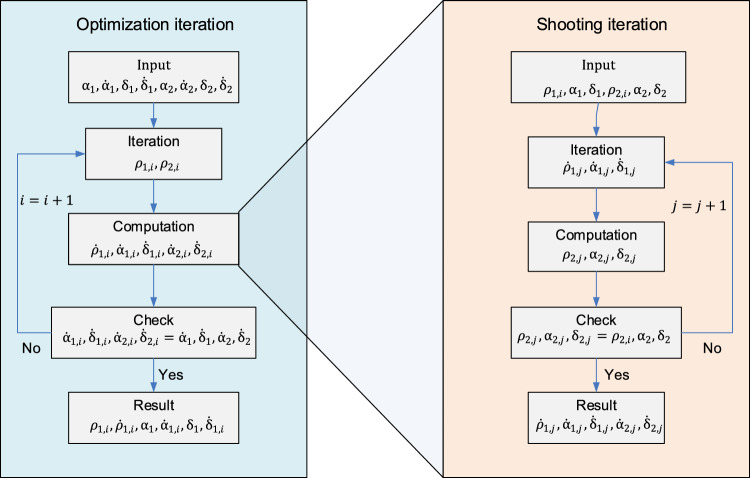
Double iteration scheme with shooting inside the optimization loop

The idea is having two nested iterations, the shooting loop being within the optimization loop (Fig. 2):

- **Shooting**: iterations over the initial velocity given the boundaries ${\rho }_{1},{\alpha }_{1},{\delta }_{1}$ and ${\rho }_{2},{\alpha }_{2},{\delta }_{2}$.
- **Optimization search**: iterations over ${\rho }_{1}$ and ${\rho }_{2}$ given ${\dot{\alpha }}_{1},{\dot{\delta }}_{1}$ and ${\dot{\alpha }}_{2},{\dot{\delta }}_{2}$ as discriminators in Eq. (3).

Note that, although both ${\rho }_{1}$ and ${\dot{\rho }}_{1}$ are free parameters, the searching procedure here is different than the one in the $({\rho }_{1}, \dot{{\rho }_{1}})$ space, because ${\rho }_{1}$ remains constant during the iteration over ${\dot{\rho }}_{1}$.

### Shooting Iterations

#### Choice of Iteration Parameters

One possible simple shooting structure in the case of Lambert problem considers position vectors ${{\varvec{r}}}_{1}({t}_{1})$ and ${{\varvec{r}}}_{2}({t}_{2})$ and iterates on the initial velocity ${{\varvec{v}}}_{1}({t}_{1})$. The positions ${\alpha }_{\text{1,2}},{\delta }_{\text{1,2}}$ at the boundary epochs ${t}_{1}, {t}_{2}$ together with the topocentric ranges ${\rho }_{1}, {\rho }_{2}$ can be easily transformed to the geocentric vectors ${{\varvec{r}}}_{1},\boldsymbol{ }{{\varvec{r}}}_{2}$ required in the Lambert model, since the position of the observing station is known. Similarly, the initial velocity is then related to the triple ${\dot{\rho }}_{1},{\dot{\alpha }}_{1},{\dot{\delta }}_{1}$. Thus, we can have shooting iterations over ${\dot{\rho }}_{1},{\dot{\alpha }}_{1},{\dot{\delta }}_{1}$ to find a solution matching the boundaries ${\rho }_{1},{\alpha }_{1},{\delta }_{1}$ and ${\rho }_{2},{\alpha }_{2},{\delta }_{2}$, as illustrated in Fig. 2 on the right.

Alternatively, since ${\dot{\alpha }}_{1},{\dot{\delta }}_{1}$ are known, one could think of including them in the shooting part as given initial condition and iterating only over ${\dot{\rho }}_{1}$, whilst matching only the condition ${\rho }_{2}$; or even using ${\dot{\alpha }}_{2},{\dot{\delta }}_{2}$ to further constrain the Newton algorithm. The former approach was followed in [40]. However, in this work, we choose the simple shooting structure with iterations over ${\dot{\rho }}_{1},{\dot{\alpha }}_{1},{\dot{\delta }}_{1}$ and no further constraints in the algorithm. One reason is that the observed ${\dot{\alpha }}_{1},{\dot{\delta }}_{1}$ have different accuracy w.r.t. ${\alpha }_{1},{\delta }_{1}$ and are difficult to be weighted in the shooting scheme. Moreover, since ${\dot{\alpha }}_{1},{\dot{\delta }}_{1}$ are set as discriminators in the optimization part, this condition is redundant in the shooting iteration and does not provide additional information.

#### Choice of Initial Guess

The shooting algorithm needs an initial guess for ${\dot{\rho }}_{1},{\dot{\alpha }}_{1},{\dot{\delta }}_{1}$ to start the iterative procedure (see Fig. 2 on the right). Good starting values can be computed considering the unperturbed Lambert case. For this part, we use the approach proposed by Izzo [14]. As seen above, there are multiple solutions to the Lambert problem. Specifically, in the multi-revolutions case, given the sense of the orbit (prograde or retrograde) and the number of revolutions between the boundary epochs, still two solutions are possible. In the context of the shooting approach, this translates into two possible solution sets of initial rates ${\dot{\rho }}_{1},{\dot{\alpha }}_{1},{\dot{\delta }}_{1}$. This means that at every iteration over ${\rho }_{1}$ and ${\rho }_{2}$, a new bifurcation arises with two new search paths and a direct method that pursues all the paths is not affordable for performance reasons. On the other hand, we know that only one solution is correct and corresponds to the absolute minimum, i.e., with the minimal deviation from the measured angular rates ${\dot{\alpha }}_{1},{\dot{\delta }}_{1}$ and ${\dot{\alpha }}_{2},{\dot{\delta }}_{2}$. We want to enforce at every iteration over ${\rho }_{1}$ and ${\rho }_{2}$, according to the measured angular rates, the selection of one solution as starting value for the shooting scheme. At any iteration step, the output of the unperturbed Lambert algorithm obviously will not match yet the measured angular rates, because ${\rho }_{1}, {\rho }_{2}$ are still not the final range values. However, the solution with values closer to the measured angular rates can be selected as starting guess, according to the following empirical criterion:

17 $${{(\Delta \dot{\alpha }}_{1})}_{1}^{2}+{{(\Delta \dot{\delta }}_{1})}_{1}^{2}+{{(\Delta \dot{\alpha }}_{2})}_{1}^{2}+{{(\Delta \dot{\delta }}_{2})}_{1}^{2}<{{(\Delta \dot{\alpha }}_{1})}_{2}^{2}+{{(\Delta \dot{\delta }}_{1})}_{2}^{2}+{{(\Delta \dot{\alpha }}_{2})}_{2}^{2}+{{(\Delta \dot{\delta }}_{2})}_{2}^{2}.$$

The $\Delta$ terms indicate the difference between the computed and measured values, whilst the bracket subscript denotes one of the two possible solutions.

#### Choice of Observation Geometry

We have to make sure that the shooting algorithm converges to the solution for the perturbed case starting from a sufficiently close unperturbed solution. This is not always the case: the correct convergence is not guaranteed when the two unperturbed solutions are very close. Referring to Fig. 1, if we consider a function $T(x)$ for given $\lambda$ and $N>0$, we see that horizontal lines (with constant $T$) intersect $T(x)$ in two different points, corresponding to two solutions. The intersections are very close to each other in proximity of the minimum value of $T(x)$. The latter corresponds to the condition of minimal time of flight for a given transfer geometry. To be sufficiently distant from that point, we consider solutions ${x}_{1}<0$ and ${x}_{2}>0$ separated by the line of minimum energy at $x=0$.

In Fig. 1 referring to the case $N=1$, we see that for $\lambda =1$, there is no analytical minimum and the intersections are on both sides of the minimum energy line. Choosing values of $\lambda$ close to 1 is a good option to obtain well-separate solutions. From the definition of $\lambda$ in Eq. (5), we know that $\lambda \approx 1$ is obtained with a small transfer angle $\theta$, i.e., a small arc between two tracklets of the observed object. The arc length can be defined with the choice of an appropriate observation geometry, e.g., with a given angular distance between the observed fields.

Furthermore, we can require a minimal distance ${\Delta }_{x}$ between the solutions: $\left|{x}_{1}-{x}_{2}\right|>{\Delta }_{x}$. The condition for $x\approx 0$ is given according to Eq. (7) when $s\approx 2a$, i.e., for highly eccentric orbits and position vectors ${{\varvec{r}}}_{1}$ and ${{\varvec{r}}}_{2}$ close to the apogee. Other $x$ values diverging from 0 allow more and more for a smaller semiperimeter $s$ and orbits with less eccentricity. Consequently, if we impose ${\Delta }_{x}$ for a given semimajor axis, only orbits with eccentricities below a certain value can be considered. However, the limiting eccentricity is in general quite high and allows in practise for an acceptable range of $\lambda$ and $x$ values. For example, for geosynchronous orbits and positions ${{\varvec{r}}}_{1}$ and ${{\varvec{r}}}_{2}$ at the apogee with $\theta \approx 15^\circ$, or $\lambda \approx 0.9$, if we require ${\Delta }_{x}\approx 0.6$, eccentricities up to 0.95 can be considered, whilst for $\theta \approx 30^\circ$ up to 0.9.

### Optimization Search

For the optimization search in the $({\rho }_{1}, {\rho }_{2})$ space, the Broyden–Fletcher–Goldfarb–Shanno (BFGS) procedure was selected in e.g., [26]. In the current implementation, we opted for a least-squares formulation of the optimization problem. In the covariance matrix ${\varvec{C}}$ in Eq. (3), the dominant term, according to [41], is the matrix ${{\varvec{C}}}_{\dot{{\varvec{z}}}}$ related to the observed rates. Since the observables $\dot{\alpha }$ and $\dot{\delta }$ are not correlated, we can treat ${\varvec{C}}$ as a diagonal matrix to a good approximation. In this case, the Mahalanobis distance ${d}_{\text{M}}$ corresponds to the classical weighted sum of the squared residuals, the latter being the difference between the observed and computed rates. This allows us to minimise ${d}_{\text{M}}$ in the frame of a least-squares problem. Besides a possible better performance in the computation of the approximate Hessian matrix, the reason for choosing this approach is that common standard implementations for least-squares estimation are available. The latter support different underlying algorithms, like e.g., Gauss–Newton or, with better performance in specific problems, Levenberg–Marquardt. The measurements ${\varvec{y}}=\left({\dot{\alpha }}_{1},{\dot{\delta }}_{1},{\dot{\alpha }}_{2},{\dot{\delta }}_{2}\right)$ have to be fitted under the estimated parameters ${\varvec{x}}=({\rho }_{1}, {\rho }_{2})$. The normal equations of the Gauss–Newton iteration scheme with the corrections ${{\varvec{\Delta}}{\varvec{x}}}^{j}$ and the measurement residuals ${{\varvec{\Delta}}{\varvec{y}}}^{j}$ are written as

18 $${{{\varvec{A}}}^{\text{T}}}^{j}{\varvec{P}}{{\varvec{A}}}^{j}{{\varvec{\Delta}}{\varvec{x}}}^{j}={{{\varvec{A}}}^{\text{T}}}^{j}{\varvec{P}}{{\varvec{\Delta}}{\varvec{y}}}^{j},$$

where ${\varvec{A}}$ is the matrix of the partial derivatives

19 $${\varvec{A}}=\left[\begin{array}{cc}\frac{\partial {\dot{\alpha }}_{1}}{\partial {\rho }_{1}}& \frac{\partial {\dot{\alpha }}_{1}}{\partial {\rho }_{2}}\\ \frac{\partial {\dot{\delta }}_{1}}{\partial {\rho }_{1}}& \frac{\partial {\dot{\delta }}_{1}}{\partial {\rho }_{2}}\\ \frac{\partial {\dot{\alpha }}_{2}}{\partial {\rho }_{1}}& \frac{\partial {\dot{\alpha }}_{2}}{\partial {\rho }_{2}}\\ \frac{\partial {\dot{\delta }}_{2}}{\partial {\rho }_{1}}& \frac{\partial {\dot{\delta }}_{2}}{\partial {\rho }_{2}}\end{array}\right],$$

and ${\varvec{P}}$ is the weighting matrix with diagonal elements $\frac{1}{{\sigma }_{i}^{2}}$. Here, ${\sigma }_{i}^{2}$ refers to the variance of the ${i}^{\text{th}}$ component of ${\varvec{y}}$. The iteration terminates when a defined convergence criterion is fulfilled, e.g., the sum of squared residuals is below a given threshold.

The Hipparchus mathematics Java library [42] was used for the above least-squares optimization. Specifically, the Levenberg–Marquardt optimizer was selected, keeping default initialisation values, with the only exception regarding the *initialStepBoundFactor*, which was set to 0.1 to limit the initial change of the Levenberg–Marquardt parameter for better convergence. This was necessary, to prevent at the beginning a bad search direction which in few cases brings temporarily to negative values of $\rho$ or object positions below the Earth surface, especially with high eccentric orbits. The choice of the initial values for ${\rho }_{1}$ and ${\rho }_{2}$ is not very critical and an average distance to a nominal orbit in the surveyed region can be adopted. For the specific case of geostationary region that we are going to analyse, we assumed ${\rho }_{\text{1,2}}=$ 36,000 km. The convergence threshold is set per default to 10^–10^ and applies to the relative difference between current and previous iteration in either the sum of squared residuals or the normed parameters solution, scaled according to the norms of the columns of the Jacobian ${\varvec{A}}$. For details, see the description of the implemented algorithm in [43].

Finally, given the initial angular positions ${\alpha }_{1},{\delta }_{1}$, the range ${\rho }_{1}$ determined in the optimization, and the rates ${\dot{\rho }}_{1},{\dot{\alpha }}_{1},{\dot{\delta }}_{1}$ found in the nested shooting iteration, the state vector at the initial epoch is calculated. As mentioned in the introduction, the orbit resulting from the correlation is usually improved with a least-squares refinement using all the single observations included in the tracklets and the output orbit of the optimization as a priori orbit. Here, the standard least-squares implementation described in [44] is employed.

## Simulation Scenario

We want to apply the developed method to simulated observations of objects in the geostationary orbit region. We take the observation scheme currently used in the survey campaigns at the ESA Space Debris Telescope in Tenerife [45] as a reference. The exposures are separated by 30 s, and on average, we can assume 5 consecutive observations. The astrometric accuracy is around 0.5″ [46] and, according to Eq. (2), this roughly corresponds to ${\sigma }_{\theta }=$ 0.2″ and ${\sigma }_{\dot{\theta }}=$ 0.02″/s. The observed fields are separated by 15° or 30° along the geostationary ring. Objects observed in the first field are re-observed later in a field shifted e.g., 15° in right ascension. The second observation is simulated after one, two, and three orbit revolutions. A smaller angle is not reasonable, because, as mentioned before, intervals multiple of the orbital period need caution. On the other hand, the argument in 5.1 suggests considering relatively small angles to guarantee the correct solution.

For the orbit determination procedure, the eccentricity of the simulated orbit is a critical parameter, as well as the observed position of the object along the orbit, since the length of the arc between the tracklets varies and thus the accuracy of the computed orbit, see, e.g., [47]. Orbits with eccentricity 0.01, 0.1, 0.3, and 0.6 were simulated, whilst the argument of perigee was so defined to observe the object in the first field at true anomalies of 0°, 60°, and 120°.

For the propagation of the simulated orbit the perturbation of Sun and Moon, a Geopotential model with coefficients up to degree and order four, and Solar Radiation Pressure (SRP) were taken into account. In the propagation the implementation in the Orekit library [39] of the Dormand–Prince integrator [48] was adopted. The computation of the SRP perturbation is based on a simple model assuming isotropic radiation and a single reflection coefficient set to 1.0 [49]. In the latter model, the area-to-mass ratio (AMR) is set to different values in m^2^/kg: 0.1, 1.0, 10.0. Including relatively high AMR values is justified by the previous studies in which objects with such characteristics have been discovered in surveys of the geostationary region [50].

## Orbit determination Results

The orbit determination procedure was tested with the above simulated observations. The tests were performed varying in each case only a subgroup of the parameter space to focus on the orbit accuracy dependency from:

- Arc length and AMR in the case where the orbit is calculated without perturbations.
- Arc length, AMR, and measurement noise.
- Arc length, true anomaly, and number of nodes in the multiple shooting.
- True anomaly, eccentricity, and angular separation of the observed fields.

The orbit accuracy is expressed as difference in position and velocity at the first observation epoch between computed and simulated orbit. The differences are represented in radial, along-track, and cross-track (RSW) components, which usually better describe characteristics of the orbit determination problem. In the following tests, we want to show the necessity to consider perturbations in the shooting step and the convergence of the method to sufficiently accurate values. This means that the computed orbit can be reliably used as a priori orbit for the decisive final orbit improvement step, mentioned above, in which the single observations, and not the attributables, are taken. In fact, the least-squares refinement of all six orbital elements is known to be critically dependent on the starting values. If the latter are too far away from the solution the algorithm might not converge. It is difficult to define a convergence radius, since it directly depends on the actual observation geometry. For more insight into the topic, see, e.g., [51]. In the performed tests, it will be indicated in which cases the least-squares refinement did not converge to a solution.

The analysis presented here does not include the application of this orbit determination method in the context of a tracklet correlation problem, although closely related to the subject. The intention is to purely evaluate the orbit computation aspect. It is clear that the procedure can be used to compute potential orbits between tracklet candidates and the correlation can be accepted according to a given criterion. However, in this context, several approaches are possible, where the gating step might be based on statistical considerations, which in general might also depend on the distance definition for the discriminators [52]. Refer to [53] for statistical analyses in terms of, e.g., true- and false-positive correlations. The evaluation of the latter is dependent on the population density, which for highly eccentric geosynchronous orbits, as for high AMR objects, is not well known.

In those tests where perturbations are considered, the same perturbation models as in the observation simulations are used. The AMR values used in the simulations are taken as known in the optimization search. We assume that an additional iteration with different AMR values can be performed and the optimal value can be found according to the best matching in the optimization search. In fact, as seen later, the algorithm still converges even without perturbation but obviously to a less correct orbit and consequently to a worse matching. In a real case, a coarse grid search with few AMR values would be performed to find a preliminary best AMR value. However, here, we want to focus more on the convergence behaviour of the main iterations rather than on this preliminary search. We know in fact that the orbit found with the preliminary best AMR is then used as a priori orbit for the final orbit improvement using single observations, and in this final step, the AMR can be taken into account as an additional estimated parameter together with the orbital elements. Thus, eventually a refined value for the AMR can be computed. It has to be mentioned that an alternative to a coarse grid search in the preliminary step is to include the AMR as estimated parameter in addition to ${\rho }_{1}$ and ${\rho }_{2}$ in the optimization search, but this would further increase the computation time.

In addition to the assumption for the AMR value, a strong limitation is given by the model assumed for the SRP perturbation. As specified above, a simplified model with isotropic radiation and a single given reflection coefficient is considered. Both these assumptions, about AMR and SRP model, relativize the analysis in terms of absolute orbit accuracy. The latter is to be considered in a best case, in which the assumed model really reflects the characteristics of the observed object. However, the reached degree of accuracy relative to the different scenarios is representative of this orbit determination problem. The validity of the procedure is not affected by the model limitations, as well as, even more important, the considerations related to the a priori orbit in the final orbit improvement. Furthermore, in the latter improvement step, even if the AMR is estimated with a simplified SRP model, the inaccuracy in the model is usually in part absorbed and compensated in the estimation of the AMR value, leading to realistic estimated orbital parameters. Here, we will not focus on the behaviour of the estimated AMR value, but we refer to, e.g., [54] for similar applications.

In the following, the results of the different tests are presented. If the parameters are not explicitly specified, default values were applied:

- Number of orbital revolutions between tracklets: 3.
- Number of shooting nodes: 5.
- Measurement noise: 0.5″ (attributable noise: 0.2″, 0.02″/s).
- AMR: 10 m^2^/kg.
- Angular separation of observed fields: 15°.
- True anomaly: 0°.
- Eccentricity: 0.01.

### Computation Without Perturbations

In this scenario, the observations are simulated with and without perturbations, but in the shooting algorithm, only a Keplerian model is applied. Table 1 shows the difference in position and velocity in radial, along-track, and cross-track components for different numbers of revolutions and AMR values. The norm of the position and velocity vectors are written in bold. Note that the observations indicated in the table with 0.0 m^2^/kg were generated without perturbations. This case illustrates the order of magnitudes of the expected error in a pure Keplerian model. Position uncertainties of several kilometres are typical for initial orbit determination based on these short tracklets. They are in agreement with values found in the literature, e.g., in [55]. We notice that in this case, the number of revolutions separating the tracklets does not influence the accuracy, rather the geometric arc length of 15° between the fields is here relevant. The position inaccuracy is in particular large in the radial component. This can be explained by the fact that angles-only and not range measurements are considered. Conversely, for the velocity, the along-track component shows the largest values, reflecting the uncertainty in the derived angular rates.

**Table 1:**
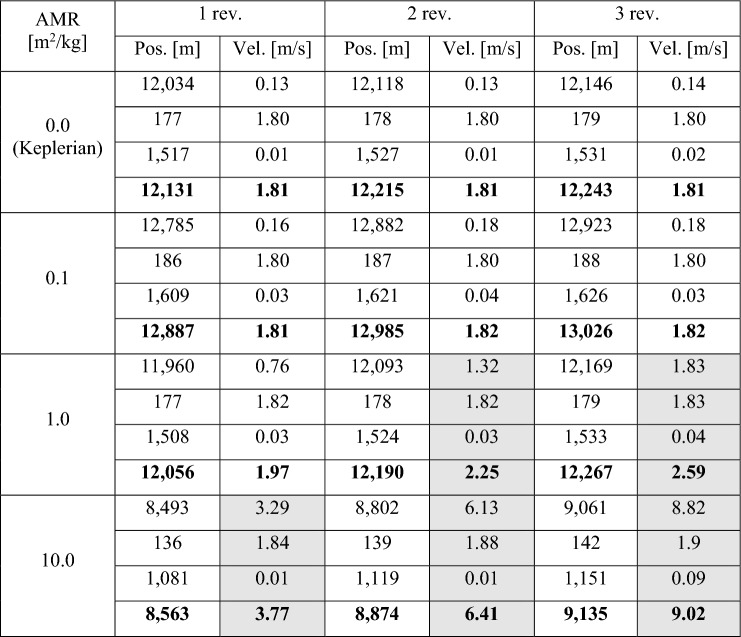
Errors for orbits computed only with a Keplerian model

The RSW components and in bold the norm of the vectors are indicated. Grey cells denote critical cases for the orbit improvement

As soon as perturbation are taken into account in the simulated measurements, a small increase of the position errors is observed in the case with AMR of 0.1 m^2^/kg. We expect with increasing number of revolutions and AMR a divergence of the resulting error, since the unperturbed model drifts apart from the simulated one over longer propagation intervals. Interestingly, whilst the position error slightly increases with the number of revolutions, the radial component becomes smaller with growing AMR, unlike the radial velocity error which turns out to be more and more significant. The two radial quantities are correlated and a better knowledge of the position is compensated by a worse accuracy in the velocity. The latter error substantially rises also with the number of revolutions, reaching about 9 m/s, one order of magnitude higher, in the case with 10.0 m^2^/kg of AMR and 3 revolutions. The table cells in grey indicate those cases in which the velocity components exceed a certain error limit and the computed orbit is not accurate enough to be used as a priori state in the least-squares orbit improvement, resulting in no final solution. In the next simulations, the developed method taking into account perturbations is applied to obviate this situation.

### Computation Including Perturbations

We now apply the developed approach including in the shooting scheme the perturbations specified above related to Earth gravitational potential, luni-solar forces, and SRP. Simulated observations again with different AMR and numbers of revolutions are considered. In addition, three different cases are treated, with no measurement noise, default noise of 0.5″, and a relatively large value of 2.5″. The latter correspond to uncertainties in the attributable of 0.2″, 0.02″/s and 1.0″, 0.1″/s, respectively.

Table 2 shows the errors of the computed orbits w.r.t. the ground truth. Here, only the norms of the error vectors in position and velocity are given. The case with no measurement noise illustrates the order of magnitude of the accuracy that can be reached. Even with no error source in the observations, the obtained orbits are only accurate to few metres in position. This very small error is introduced in the actual computation procedure, due to tolerances in numerical integration and optimization. The simulations with default noise (0.2″, 0.02″/s) can be compared directly with the results of the previous tests in Table 1, which shows the same dependency from AMR and number of revolutions, with the only difference that perturbations were not included in the orbit determination. Contrary to the previous case, here, the error in position and velocity are relatively stable around 12,000 m and 1.81 m/s. The increase in AMR and arc length does not essentially affect the accuracy. Since the velocity inaccuracy remains at acceptable levels, the resulting orbit can be used as starting point to successfully perform the final orbit improvement. The consequence of observations with larger noise 1.0″ and 0.1″/s directly results in bigger errors, proportional to the noise increase. Interestingly, although the orbit inaccuracy exceeds by far the largest values in Table 1, no problem was encountered using these computed orbits as a priori values. The major difference here is that the single simulated measurements have also a broader distribution according to the higher noise and the convergence radius for the initial guess increases correspondingly.

**Table 2:** Errors for orbits computed with perturbations

Noise	AMR	1 rev		2 rev		3 rev	
		Pos. [m]	Vel. [m/s]	Pos. [m]	Vel. [m/s]	Pos. [m]	Vel. [m/s]
No noise	0.1	0.53	0.0013	0.71	0.0031	2.50	0.0069
	1.0	5.30	0.0043	7.88	0.012	11.25	0.018
	10.0	22.96	0.022	32.27	0.015	38.35	0.026
0.2″ 0.02″/s	0.1	12,145	1.81	12,227	1.81	12,253	1.81
	1.0	12,137	1.81	12,208	1.81	12,228	1.81
	10.0	12,086	1.81	12,160	1.81	12,167	1.81
1.0″ 0.1″/s	0.1	121,011	18.09	121,823	18.09	122,102	18.10
	1.0	120,939	18.08	121,693	18.08	121,923	18.08
	10.0	120,017	17.97	121,225	18.03	118,840	17.74

Only the norms of position and velocity error vectors are indicated

We give indicative values of the accuracy obtained after the final orbit improvement: for example, in the case with one revolution and default noise and AMR, the final orbit has errors of 2497 m and 1.22 m/s in position and velocity, respectively, owing to the better statistics related to the single observations. This accuracy is compatible with observation schemes which aim at orbital catalogue build-up and maintenance [56].

### Dependence on Orbit Shape and Observation Fields

In the previous tests, we considered orbits with low eccentricity. Here, we investigated whether a change in the shape of the orbit and the observation at different places along the orbit has an influence on the accuracy. The default simulation values, as specified at the beginning of the section, are assumed, but with variations in eccentricity and true anomaly. The argument of perigee is chosen, so that the object is observed in the first field at true anomalies of 0°, 60°, and 120°. In addition, a different angular separation of 30° between the observed fields was also tested.

Table 3 shows the obtained results expressed as norms of the position and velocity error vectors in [m] and [m/s]. The increase in the eccentricity results in a general improvement of the accuracy in both position and velocity, owing to a different geometry of the problem. The change in the anomaly of the observed object along the orbit causes also a variation in the accuracy. In general, it seems that for higher anomaly values, the error rises. However, the described trends are not followed consistently in all the cases. We see that, e.g., for the case at 120°, the position error remains very similar with 0.1 and 0.3 eccentricity, whilst the velocity error increases and reaches for 0.6 eccentricity a value higher than the one at 0.01 eccentricity. On the other hand, the errors obtained with 30° field separation vary consistently with the ones in the 15° simulations. Although in the two scenarios, the arc length quite differs, the accuracy is very similar. This can be taken into account in the choice of the observation strategy. In general, we can state that changing the shape of the orbit and the geometry of the problem does not essentially affect the quality of the orbit determination. The obtained orbits successfully provided valid a priori states to the final least-squares refinement.

**Table 3:** Position and velocity errors in [m] and [m/s] for various eccentricities, true anomalies, and field separations (15°, 30°)

*e*	15°						30°					
	0°		60°		120°		0°		60°		120°	
	Pos	Vel	Pos	Vel	Pos	Vel	Pos	Vel	Pos	Vel	Pos	Vel
0.01	12,247	1.81	12,420	1.82	12,760	1.85	12,199	1.82	12,363	1.84	12,700	1.86
0.1	9449	1.60	10,731	1.73	14,064	1.96	9424	1.62	10,628	1.75	13,871	1.98
0.3	4669	1.16	8935	2.51	14,662	2.11	4693	1.17	7468	1.53	13,893	2.19
0.6	968	0.92	943	1.50	6,886	2.06	988	1.22	1258	1.20	5575	2.22

### Dependence on the Number of Shooting Nodes

In this analysis, we want to investigate the dependence of the orbit accuracy from the number of nodes assumed in the multiple shooting method. The tests were conducted with different numbers of revolutions, since we expect the arc length to have an influence on the accuracy of the shooting method. Over longer intervals, the propagated orbit possibly diverges more and more from the actual one. A subdivision of the overall arc into smaller propagation intervals might be of advantage. We assumed an eccentricity of 0.6 and the anomaly value of 120° to evaluate a scenario in which the orbit propagation might be more demanding. Table 4 shows the results of the simulations. Position and velocity errors are given in [m] and [m/s]. We notice that the errors do not significantly change for higher numbers of revolutions or varying the numbers of nodes. Longer arcs do not affect the shooting algorithm performance and there is no need to reduce the length of the multiple arcs. There are some slightly smaller values for ten nodes for one and two revolutions, as well as for 15 nodes in the three revolutions case. However, the differences are relatively small and there is no clear trend in the overall sequence of values. These results suggest that even a lower number of nodes is acceptable. Although not reported here, other tests have shown that even with only 1 node, the accuracy remains essentially the same. On the contrary, the computation time augments together with the amount of the subdivisions. Since the total arc length is the same, the time associated purely with the propagation remains unchanged, but the size of the solution matrix becomes larger. As an example, for the one revolution case, adding one node more causes an overhead of about 20% computation time. Since the propagation is the critical part, dividing the whole arc in smaller intervals makes only sense if the calculation within the single subintervals is run in parallel. Indeed, in our specific orbit determination problem, since the accuracy is not affected, parallel computing of the multiple shooting scheme would provide a considerable advantage in terms of computation time, which is a critical point.

**Table 4:** Position and velocity errors in [m] and [m/s] for different numbers of revolutions and shooting nodes

	5 nodes		10 nodes		15 nodes		20 nodes		30 nodes		40 nodes	
	Pos	Vel	Pos	Vel	Pos	Vel	Pos	Vel	Pos	Vel	Pos	Vel
1 rev	6820	1.96	6,664	1.97	6,793	1.96	6788	1.96	6844	1.95	6828	1.95
2 rev	6745	1.98	6,577	2.0	6,879	1.97	6663	1.99	7334	1.92	6953	1.96
3 rev	6151	2.0	7,056	1.95	5,644	2.12	6465	2.0	6483	2.02	7153	1.95

Eccentricity of 0.6 and anomaly of 120° are assumed

## Computation Time

We want to estimate the computation time needed by the orbit determination. The latter is dependent from the number of revolutions considered between the observations and the consequent total propagation time. Table 5 shows the performance of the algorithm as a function of the number of revolutions and with one shooting node. The software ran on a 3.2 GHz Intel Core i5 CPU. The first row indicates a case using default simulation values, as specified at the beginning of this section, but no perturbations. The required time is small, around 7 s, even for more revolutions. The second row corresponds to tests with default simulation values including perturbations. Since the perturbations are now included the necessary time increases and it is approximately proportional to the propagation interval. For three revolutions, about 5 min have to be taken into account. Note that most of the processing time is required by the numerical integration scheme which takes more than 90% of the total. For specific orbits especially with higher eccentricity, the elapsed time seems to be even greater. The last row in Table 5 displays the results with perturbations using default simulation values but with eccentricity of 0.6 and represents approximately those cases where the highest processing time is requested. We see that the time does not grow proportionally to the number of revolutions, and therefore, highest values up to more than 45 min are reached. If we consider the three revolutions interval a realistic approach, we have required times between 5 and 15 min, which depending on the application might be still acceptable.

**Table 5:** Computation time for different numbers of revolutions

	1 rev	2 rev	3 rev	4 rev
w/o perturb	7.0 s	7.5 s	7.7 s	7.8 s
With perturb	107 s	255 s	311 s	398 s
0.6 eccentr	220 s	421 s	1075 s	2792 s

In all three cases, the default simulation values, specified at the beginning of this section, were considered. In the first, a model without perturbations is applied, in the second with perturbations, whilst in the last with perturbations and default values up to the eccentricity which differs

## Conclusions

In this work, a method for orbit determination based on the optimised boundary value formulation in [17] is proposed. The original method applies to passive optical observations and makes use of an optimization procedure together with a Lambert solver to approach the boundary value problem. In this novel approach, we extended the method to include orbital perturbations by means of a shooting scheme. The latter replaces the Lambert solver in the optimization and includes the perturbations in the numerical propagation of the orbit. In particular, a multiple shooting algorithm was proposed, which divides the propagation arc in multiple subintervals. In the shooting procedure, a multi-variable Newton method was adopted to search for the correct boundary solution. Since the solution of the Lambert problem is not unique, additional constraints were proposed to identify the correct orbit. These conditions partly depend on the angular distance of the observed fields, which can be tailored in the observation strategy to guarantee a proper convergence of the algorithm.

Simulations of observation scenarios for the geostationary region were performed. Different values of arc length, noise, perturbations, number of shooting nodes, and orbital shape were considered. The computed orbits were compared with the ground truth of the simulations and the difference was evaluated to assess the convergence and the accuracy of the method. It was shown that, especially for the perturbation due to the solar radiation pressure (SRP) over two or three orbit revolutions and assuming area-to-mass ratios (AMR) between 1.0 and 10 m^2^/kg, the proposed method provides sufficiently accurate orbits which can be successfully used as a priori values in an ordinary least-squares orbit improvement procedure. On the contrary, with a pure Keplerian propagation, the accuracy of the calculated a priori orbit was not in all test cases enough for the least-squares process to converge. From the orbit improvement step, accuracies were obtained, which allow the object to be identified in subsequent observations and to be catalogued, according to the literature. Simulations with different observation fields and orbit eccentricity showed no significant worsening of the accuracy. The results obtained in tests with larger measurement noise also remain within the valid range of values for a reliable orbit improvement.

The dependence of the efficiency of the multiple shooting method on the number of subintervals was investigated. The orbit quality barely depends on the latter number, even in scenarios with longer propagation arcs of two or more orbit revolutions. Since the multiple arcs solution has a slight overhead in terms of processing time, it is suggested to keep the number of arcs low. However, the analysis of the overall computation time required by the proposed method shows that most of the time is spent in the orbit propagation, especially when the solar radiation pressure is taken into account. Thus, performing the propagation within the single subintervals in parallel processes would be computationally more efficient. The required time is indeed a critical aspect: in the typical simulated scenarios running on a 3.2 GHz Intel Core i5 CPU, the orbit determination process took in the order of 100 s in the case with one orbit revolution, and up to 300 s for three revolutions. Whilst this is still acceptable, there were cases with more than 15 min for the same number of revolutions. Due to the high time consumption, the parallelization of the process would be a very appealing approach.

The developed method was applied to observations in the geostationary regime, in the context of debris survey scenarios and specific object populations, with, e.g., high values of area-to-mass ratio. However, the studied procedure might be used also in the other orbital regimes, given that the observation strategy is compatible with the object’s visibility conditions and specifically with the identified constraints in the Lambert problem. Other perturbations might be important in other regimes, e.g., drag forces. However, the convergence behaviour and the attainable accuracy cannot be guaranteed on other orbital regions and would require further investigation.

An important step in future work is certainly to evaluate the performance of the method with real observations coming from applied survey strategies. Moreover, further aspects related to the SRP model and the AMR estimation need to be more critically evaluated in terms of accuracy and limitations in the overall orbit determination procedure. The method shall be integrated in an existing tracklet correlation procedure and at that point, having a large number of orbits to be calculated, the algorithm performance will have to be possibly optimised by means of parallel computing.

## Data Availability

No datasets were generated or analysed during the current study.
